# In Hospital Stroke Mortality: Rates and Determinants in Southwestern Saudi Arabia

**DOI:** 10.3390/ijerph15050927

**Published:** 2018-05-07

**Authors:** Adel A. Alhazzani, Ahmed A. Mahfouz, Ahmed Y. Abolyazid, Nabil J. Awadalla, Khaled Katramiz, Aesha Faraheen, Shamsun Nahar Khalil, Razia Aftab

**Affiliations:** 1 Department of Internal Medicine, College of Medicine, King Khalid University, Abha 61421, Saudi Arabia; alhazzani@kku.edu.sa; 2 Department of Family and Community Medicine, College of Medicine, King Khalid University, P.O. Box 641, Abha 61421, Saudi Arabia; drzizous2000@yahoo.com (A.Y.A.); njgirgis@yahoo.co.uk (N.J.A.); draeshasiddiqui@gmail.com (A.F.); shamsun2003@gmail.com (S.N.K.); drraziaaftab@gmail.com (R.A.); 3 Department of Epidemiology, High Institute of Public Health, Alexandria University, Alexandria 21511, Egypt; 4 Department of Community Medicine, College of Medicine Mansoura University, Mansoura 35516, Egypt; 5 Department of Neurology Section, Aseer Central Hospital, Saudi Arabia, Abha 21411, Saudi Arabia; kneuro76@hotmail.com

**Keywords:** first-time stroke, in-hospital mortality, case fatality, determinants, Saudi Arabia

## Abstract

*Objectives:* The present study analyzed in-hospital first-time stroke mortality in southwestern Saudi Arabia over one-year to assess the in-hospital stroke case fatality rate, mortality rate and explore the factors associated with in-hospital stroke mortality. *Study Design*: Hospital based follow-up study. *Methods:* First-time stroke patients admitted to all hospitals in Asser region over one-year period (January through December 2016) were included in the study. Data about personal characteristics, pre-stroke history and clinical criteria, on admission clinical criteria, in-hospital complications and survival status were collected. The last reported Aseer region population was used to calculate age and sex stroke mortality rate per 100,000 population/year. Hazard ratios (HR) and concomitant 95% confidence intervals (95% CI) were computed using multivariate Cox regression survival analysis. Kaplan-Meier curve survival analysis for stroke patients were plotted. *Results:* A total of 121 in-hospital deaths out of 1249 first-time stroke patients giving an overall case fatality rate (CFR) of 9.7%. Non-significant difference with gender and age were observed in CFR. Overall, in-hospital stroke mortality rate was 5.58 per 100,000/year. Males and elders showed a significantly higher mortality rates. Multivariable Cox regression analyses revealed pre-stroke smoking (HR = 2.36), pre-stroke hypertension (HR = 1.77), post-stroke disturbed consciousness (HR = 6.86), poor mobility (HR = 2.60) and developing pulmonary embolism (HR = 2.63) as significant predictors of in-hospital stroke mortality. *Conclusions*: In Southwestern Saudi Arabia, the in-hospital stroke mortality rate is higher in men and increases with aging. The prognosis of acute stroke could be improved by smoking cessation, better control of hypertension and prevention of in hospital complication particularly pulmonary embolism.

## 1. Introduction

In the latest update on stroke mortality by WHO, it is reported that the highest rate of stroke mortality is represented by the middle and low income countries [1]. In industrial countries, the in-hospital stroke fatality rate is 3–11% [2,3]. Whereas studies from developing countries it ranged from 7–15% [4,5].

Stroke mortality is known to be associated with patient and hospital related aspects. Studies showed that increased probability of in-hospital mortality is associated with increasing age [6,7,8] and significantly higher among women as compared to men [6,9].The hospital related factors include, availability of a stroke care unit and the patient’s residence related to the hospital [8].

A review article of stroke epidemiology in the middle east region reported an overall in- hospital stroke mortality rate of 8–28% [10]. A recent study in Saudi Arabia in Jeddah in 2016 reported an overall mortality of around 27% and emphasized that there was no decrease in stroke mortality over the previous 5-year period [11]. Globally, it is expected that the impact of this condition will increase in the coming years [12,13,14]

In Aseer region, southwestern Saudi Arabia, there is a scarcity of publications about in-hospital stroke mortality. Exploring extent of in-hospital stroke mortality and determinants in the region might be helpful in defining future resource allocations and health care policies that might impact or improve care of individuals of stroke. The aim of the present work was to study in-hospital first-time stroke mortality in the region, to assess the case fatality rate and explore the factors associated with in-hospital stroke mortality.

## 2. Methods

### 2.1. Settings

The present study is a hospital based follow-up study.

### 2.2. Study Area

The Aseer Region is located in the southwest of Saudi Arabia. The last reported population is 2,166,983 [15]. A network of 242 primary health care centers, 12 secondary care hospitals and one tertiary hospital (Aseer Central Hospital) deliver health care services in the region. The ethical project approval identification code is REC# 2014-03-08.

### 2.3. Data Collection

The present study was performed over one full year period (1st of January throughout 31 of December 2016). The study included all first-time hospitalized stroke cases in Aseer region. Neurologists examined the diagnosis of the study cases based on Saudi Ministry of Health criteria (MOH Pocket Manual in Critical care and ICD-10). They included conditions of focal neurological deficit (either cerebral infarction or hemorrhage) established by CT or MRI. Glasgow scale was used to assess level of consciousness on admission. Thorough history taking with relatives was done to exclude recurrent stroke cases.

Acute stroke cases were admitted only to secondary or tertiary care hospitals. If cases were transferred for any reason between different hospitals in the region, double counting was avoided. Patients outside the region were not included and no cases were referred outside the region. Data about personal characteristics, pre-stroke history and clinical criteria, on admission clinical criteria, in-hospital complications and survival status were collected. End point of hospital mortality was at 90 days.

### 2.4. Statistical Analysis

Last reported Aseer region population was used to calculate age and sex stroke mortality rate per 100,000 population/year. In-hospital stroke case fatality rate was calculated. Analysis was performed using SPSS version 22. Proportions (Case fatality rate and mortality rate per 100,000 persons) and associated 95% confidence intervals (95% CI) were calculated. Hazard ratios and their 95% CI were estimated using multivariate Cox regression survival analysis. Kaplan-Meier curve survival analysis for stroke patients were plotted.

## 3. Results

Out of 1249 first-time hospitalized stroke cases in Aseer region, the study reported 121 in-hospital deaths giving an overall case fatality rate of 9.7% (95% CI: 8.1–11.5). The average length of hospital stay was 10.67 ± 17.38 days and a median of 7 days. Recurrent stroke cases amounted to 312 and were not included in the study.

Table 1 shows the in-hospital case fatality rate (CFR) and concomitant 95% confidence intervals (95% CI) by age group and gender. The CFR among males aged <40 years amounted to 14.3% (95%CI: 7.1–24.7). The CFR among females in the same age group was 10.9% (95%CI: 6.1–17.5). There was no overlapping in the confidence intervals indicating absence of significant gender difference. Similar non-significant differences by gender were observed in other age groups (40–49 years, 50–59 years, 60–69 years, 70–79 years and 80+ years). Overall, the CFR increased from 10.9% (95% CI: 6.1–17.5) among those aged <40 years to reach 15.9% (95%CI: 12.1–20.4). Yet, no statistical significance was found. Similar trend of non-significant differences by age groups were observed among both sexes.

**Figure 1:**
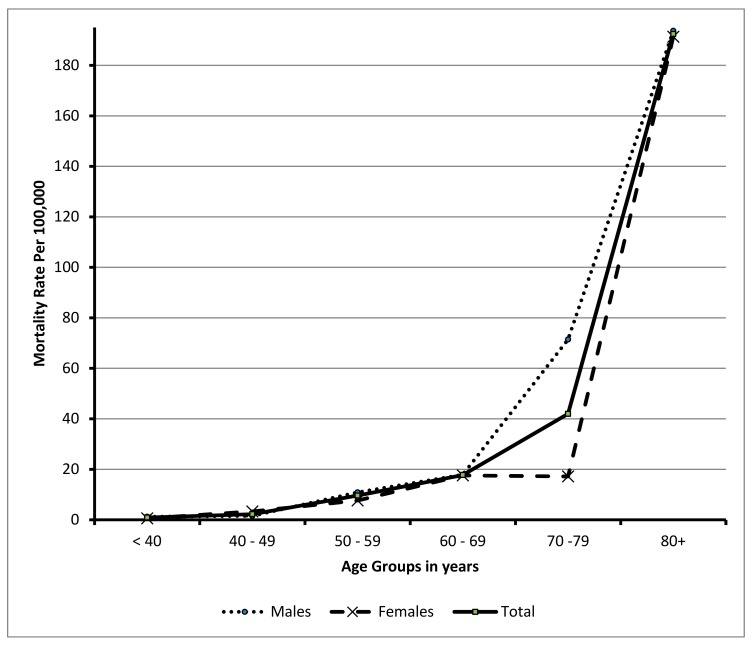
In-hospital stroke mortality rate per 100,000 population by different Age groups and gender.

Table 2 and Figure 1 show the in-hospital first-time stroke mortality rate (per 100,000 populations) and concomitant 95% Confidence Intervals (95% CI) by age groups and gender, giving a total mortality rate of 5.58 per 100,000 population (95% CI: 5.55–5.61). The mortality rate raised from 0.91 (95% CI: 0.89–0.93) among those aged less than 40 years to reach 192.46 (95% CI: 187.7–197.3). The figure shows an increase trend by age group with a significant difference. Similar significant trend was detected in males and females. Comparing both sexes, a significant variation was found in each age group.

Table 3 shows the in-hospital first-time stroke mortality predictors (Cox regression Hazard Ratio) and concomitant 95% confidence intervals (95% CI). Regarding socio-demographic variables, females had Hazard ratio of 1.317 compared to males. Yet this ratio is not statistically significant (95% CI: 0.856–2.025). The rest of the socio demographic variables (age, altitude and nationality) were not statistically different.

**Figure 2:**
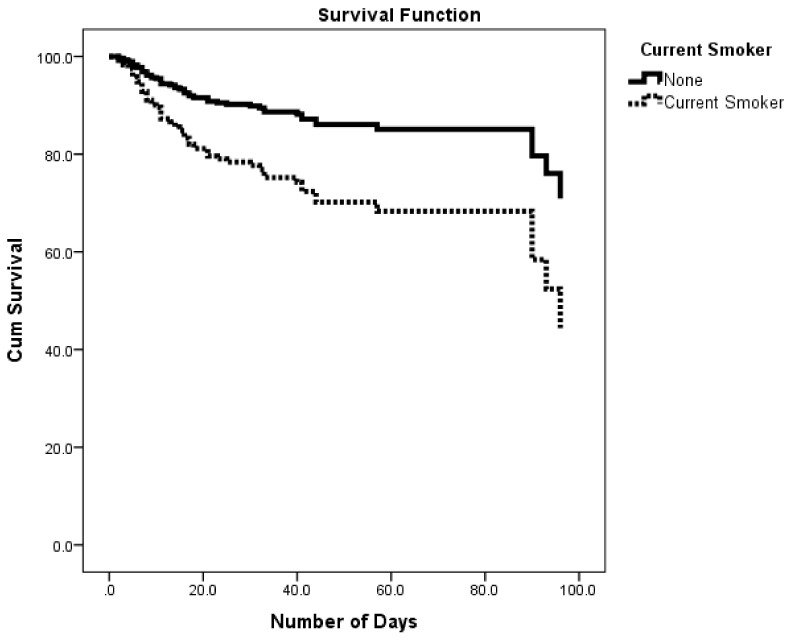
Survival rates for in-hospital stroke patients by current smoking status.

As for family and clinical history among stroke patients (Table 3) current smokers had significantly higher risk of in-hospital mortality (HR = 2.363, 95% CI: 1.202–4.643). Kaplan-Meier survival analysis for the effect of smoking on cumulative survival of stroke patients revealed a significantly lower cumulative survival percentage among smokers compared to non-smokers (Figure 2). Similarly, hypertensive patients had significantly higher risk of in-hospital mortality (HR = 1.776, 95% CI: 1.056–2.988). On the contrary, other co morbidities (diabetes, hypercholesterolemia, obesity and Atrial fibrillation) were not statistically different.

**Figure 3:**
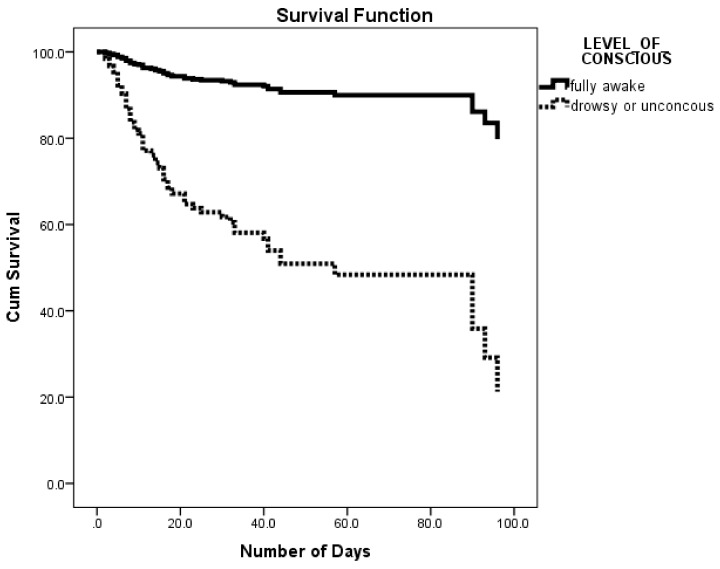
Survival rates for in-hospital stroke patients by level of consciousness.

Regarding clinical condition on admission (Table 3), altered level of consciousness on presentation had significantly higher risk of in-hospital mortality (HR = 6.861, 95% CI: 3.42–11.941). Kaplan-Meier survival analysis for the effect of level of consciousness on admission on cumulative survival of stroke patients revealed a significantly lower cumulative survival percentage among drowsy and unconscious patients compared to fully awake patients (Figure 3). Similarly, immobile patients on admission had significantly higher risk of in-hospital mortality (HR = 2.605, 95% CI: 1.559–4.352). On the other hand, hospital arrival time was not significant.

**Figure 4:**
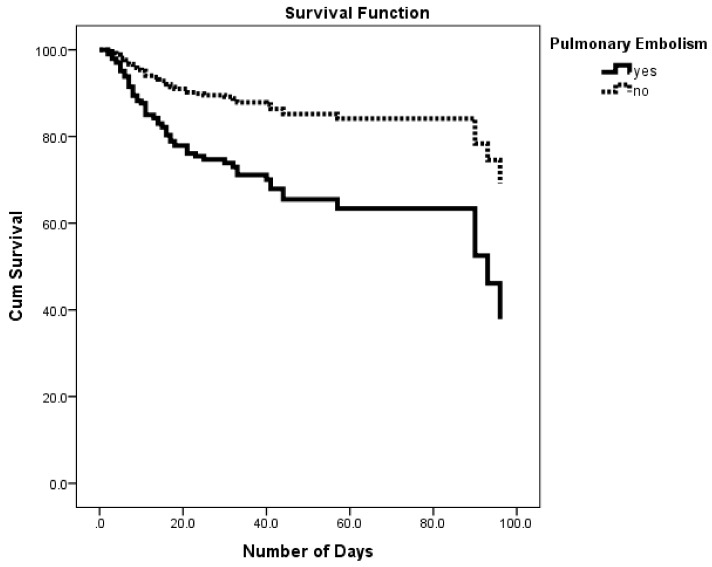
Survival rates for in-hospital stroke patients by pulmonary embolism.

As for in-hospital complications (Table 3), patients who developed pulmonary embolism had significantly higher risk of in-hospital mortality (HR = 2.636, 95% CI: 1.516–4.585). Kaplan-Meier survival analysis for the effect of developing pulmonary embolism on cumulative survival of stroke patients revealed a significantly lower cumulative survival percentage among those who developed pulmonary embolism compared to those who did not (Figure 4). On the other hand, development of deep vein thrombosis and pneumonia were not significant.

## 4. Discussion

The current study reported 121 in-hospital deaths out of 1249 first-time stroke patients admitted to the study hospitals in Asser region, Saudi Arabia during the study period from the first of January 2016 till 31 December 2016 giving an overall case fatality rate (CFR) of 9.7%. This figure was lower than that reported by studies conducted in sub-Saharan Africa which ranged from 18.8% in Nigeria to 27% in Gambia [16]. The current figure was also lower than that reported CFR at 30-days in Poland (29%) [17] and 28-days in Brazil (12.6%) [18]. In the Middle east region the overall in-hospital CFR of stroke was 8–28% [10]. On the other hand, in industrial countries, the in-hospital CFR among stroke patients ranged from 3–11% [2,3]. The current figure was slightly lower than that reported in Jeddah, Saudi Arabia (11.1%) [11].

In the current study, the CFR was not significantly affected by gender in each age group. Also, the overall CFR and CFR in both sexes showed a non-significant increase trend by age. Our results were in agreement with a previous review article that showed no relation between sex and overall CFR [19]. However, our study was different from a study in Tanzania, which reported a significant increase of case fatality over the age of 65 years [16].

The results of the present study, reported a total in-hospital stroke mortality rate of 5.58 per 100,000 population. This is the first study to report the in-hospital mortality rate in Saudi Arabia and it gives the baseline figure to evaluate the subsequent changes in the trend of stroke mortality. The in-hospital stroke mortality rates differ between nations. These differences may be attributed to incidence, case fatality, or both [18]. The mortality rate revealed by the present study was considered low compared with that reported in Lithuania (572.1/100,000 population) [20]. The explanations of this difference may include; firstly, the present study included only the in-hospital deaths, secondly, the low incidence of stroke in southwestern Saudi Arabia (57.64 per 100,000person per year) [21] and the higher case fatality rate in Lithuania (20.1%) [20]. Similarly, our results were lower than the seven countries that reported their stroke mortality rates to WHO namely; Russian Federation, Ukraine and Belarus, Turkmenistan, Kazakhstan, Seychelles and Oman. Their figures ranged from 26 In Oman to 233/100,000 population in Russian Federation [22].

The present study revealed a significant increase in mortality rates with age. This was in accordance with the results of a recent systematic review for the studies conducted in the Middle East, which found a significant positive correlation between age and the mortality rate [10]. Furthermore, the current study observed a significant higher mortality in males compared with females in each age stratum. Although the current study failed to find a significant difference between males and females in case fatality rates, however the existing higher incidence of stroke in males compared with females reported in southwestern Saudi Arabia [21] could explain this gender difference in mortality rate.

In the current study pre-stroke smoking, pre-stroke hypertension, post-stroke disturbed consciousness, poor mobility and developing pulmonary embolism were significant risk factors of in-hospital stroke mortality.

The present study revealed that smoking status was significant risk factor for in-hospital mortality. This result was supported by findings from both developing and developed countries including; Tanzania [16], Cameron [23], Malaysia [24], Canada [25], China [26] and USA [27].

Also, our study confirmed the positive association of pre-stroke hypertension and risk of in hospital stroke mortality. In Poland the untreated pre stroke hypertension was important predictor of 1-year mortality from stroke [17]. Similarly a Chinese study has supported the significant association between high blood pressure on admission and mortality from stroke [28]. Additionally, elevated blood pressure was a risk of one month stroke mortality in sub-Saharan African [23].

According to our results, disturbed level of consciousness on admission was a significant predictor of in-hospital mortality. A similar result was observed in studies conducted in Tanzania study [16],Cameron [23], Poland [17] and Switzerland [29].

Furthermore, the current study revealed that poor mobility on admission was significantly associated with higher risk of in-hospital mortality. Studies from Tanzania, Gambia, Nigeria, Congo and Cameroon had reported the same finding [16]. Also, in Poland immobility and immobility co-morbidities contributes to high stroke case fatality [17]. There is some evidence that immobility following stroke is not only an indicator of stroke severity but also it increases the likelihood of complications like pulmonary embolism [17]. This was confirmed in our study which detected post stroke pulmonary embolism as a significant predictor of in-hospital mortality. Pulmonary embolism was the cause for more than one eighth of early deaths after stroke and the use of prophylactic anticoagulant to prevent venous thromboembolism is highly recommended to reduce the risk of early stroke deaths [30].

The present study has several strengths. The study has the merit of being the first to report the in-hospital mortality in Saudi Arabia. The prospective study design, the relatively large number of study subjects and the complete follow-up for mortality are main strengths of the study. It gives the baseline figure to evaluate the subsequent changes in the trend of stroke mortality regionally and nationwide. The study identified some predictors of stroke mortalities that can be used to improve the survival of stroke patients. The revealed data are important to improve stroke management paradigm in hospitals.

Limitations of the study include the lack of detailed analysis of mortality by stroke type. TPA medication use in acute stroke management was unaccounted for in the analysis. Similarly, the present study may not reveal the overall picture of mortality in the population as we only looked at those admitted to the hospital.

## 5. Conclusions

In Southwestern Saudi Arabia, the case fatality rate is comparable with reported data from Saudi Arabia and some developed and developing countries. The in-hospital stroke mortality rate is higher in men and increases with aging. The prognosis of acute stroke could be improved by smoking cessation, better control of hypertension and prevention of in hospital complication particularly pulmonary embolism. There is a need to develop more effective stroke management services in south-western Saudi Arabia.

## Figures and Tables

**Table 1:** In hospital first time stroke case fatality rate (%) and concomitant 95% Confidence Intervals (95% CI) by age groups and gender.

Age	Males			Females			Total		
	No. Died	CFR (%)	95% CI	No. Died	CFR (%)	95% CI	No. Died	CFR (%)	95% CI
<40–	10	14.3	7.1–24.7	4	6.8	2.2–15.5	14	10.9	6.1–17.5
40–49	3	3.9	1.0–10.4	4	8.5	2.4–20.4	7	5.7	2.3–11.4
50–59	11	9.0	4.8–15.1	5	8.6	2.9–19.0	16	8.9	5.2–14.0
60–69	8	5.4	2.6–10.1	7	8.9	3.6–17.4	15	6.6	3.8–10.7
70–79	14	8.4	4.7–13.7	4	3.9	1.1–9.6	18	6.7	4.0–10.3
80+	25	12.9	8.5–18.4	26	20.5	13.8–28.5	51	15.9	12.1–20.4
Total	71	9.1	7.3–11.3	50	10.6	7.9–13.7	121	9.7	8.1–11.5

No Significant differences in case fatality rate by gender (based on 95% CI in different age groups—All the two confidence intervals overlap).

**Table 2:** In-hospital first-time stroke Mortality rate (per 100,000 populations) and concomitant 95% Confidence Intervals (95% CI) by age groups and gender.

Age Group	Males		Females		Total	
	Deaths/Pop.	Mortality Rate per 100,000 (95%CI)	Deaths/Pop.	Mortality Rate per 100,000 (95%CI)	Deaths/Pop.	Mortality Rate per 100,000 (95%CI)
<40	10/813,170	1.230 (1.20–1.260) *	4/719,814	0.560 (0.550–0.561) *	14/1,532,984	0.910 (0.890–0.930)
40–49	3/192,850	1.555 (1.500–1.610) *	4/120,629	3.310 (3.220–3.420) *	7/313,479	2.230 (2.180–2.290)
50–59	11/101,192	10.870 (10.680–11.060) *	5/65,405	7.644 (7.440–7.850) *	16/166,597	9.604 (9.460–9.750)
60–69	8/44,736	17.882 (17.530–18.240)	7/39,806	17.585 (17.210–17.960)	15/84,542	17.742 (17.490–18.000)
70–79	14/19,603	71.417 (70.78–72.05) *	4/23,280	17.18 (16.70–17.67) *	18/42,883	41.97 (38.0–53.0)
80+	25/12,909	193.66(186.9–200.6)	26/13,589	191.33 (184.7–198.0)	51/26,498	192.46 (187.7–197.3)
Total	71/1,184,460	38.491 (38.270–38.710) *	50/982,523	5.10 (5.050–5.130) *	121/2,166,983	5.584 (5.550–5.610)

Pop. = Population in Aseer region by age in mid-2016 (demographic survey 2016, The Saudi General Authority for Statistics)*.* * Significant differences in mortality rate by gender based on 95% CI (if the two confidence intervals do not overlap).

**Table 3:** In-hospital First-time Stroke Mortality Predictors (Cox regression Hazard Ratio) and concomitant 95% Confidence Intervals (95% CI).

Variables	Hazard Ratio (HR)	95.0% CI	
		Lower	Upper
**Socio-Demographic:**			
Age: 70+ vs. < 70 years	0.950	0.601	1.500
Gender: Females vs. Males	1.317	0.856	2.025
Altitude: Low vs. High	1.220	0.778	1.913
Nationality: Non-Saudi vs. Saudi	1.644	0.905	2.985
**History:**			
Family History of stroke: Yes vs. No	0.646	0.277	1.511
Diabetes Mellitus: Yes vs. No	0.721	0.429	1.210
*Hypertension: Yes* vs. *No* *	1.776	1.056	2.988
Hypercholesterolemia: Yes vs. No	1.208	0.738	1.977
*Current Smoker: Yes* vs. *No* *	2.363	1.202	4.643
Obesity: Yes vs. No	1.322	0.884	1.977
Atrial Fibrillation: Yes vs. No	1.016	0.531	1.943
**Clinical Condition on Admission:**			
*Level of consciousness: Drowsy or unconscious* vs. *alert* *	6.861	3.942	11.941
*Mobility: Immobile* vs. *mobile* *	2.605	1.559	4.352
Hospital arrival time: 3 h+ vs. < 3 h	1.319	0.827	2.104
**In-hospital compilations:**			
Deep Vein Thrombosis: Yes vs. No	0.873	0.197	3.879
*Pulmonary Embolism: Yes* vs. *No* *	2.636	1.516	4.585
Pneumonia: Yes vs. No	0.700	0.447	1.096

* Significant Hazard ratio.

## References

[1] Thrift A.G., Thayabaranathan T., Howard G., Howard V.J., Rothwell P.M., Feigin V.L., Norrving B., Donnan G.A., Cadilhac D.A. (2017). Global stroke statistics. Int. J. Stroke.

[2] Koennecke H.-C., Belz W., Berfelde D., Endres M., Fitzek S., Hamilton F., Kreitsch P., Mackert B.-M., Nabavi D., Nolte C. (2011). Factors influencing in-hospital mortality and morbidity in patients treated on a stroke unit. Neurology.

[3] Candelise L., Gattinoni M., Bersano A., Micieli G., Sterzi R., Morabito A., Group T.P.S. (2007). Stroke-unit care for acute stroke patients: An observational follow-up study. Lancet.

[4] Rocha M.S.G., Almeida A.C.F., Abath Neto O., Porto M.P., Brucki S.M.D. (2013). Impact of stroke unit in a public hospital on length of hospitalization and rate of early mortality of ischemic stroke patients. Arq. Neuro-Psiquiatr..

[5] Borhani-Haghighi A., Safari R., Heydari S.T., Soleimani F., Sharifian M., Kashkuli S.Y., Khayatghuchani M.N., Azadi M., Shariat A., Safari A. (2013). Hospital mortality associated with stroke in Southern Iran. Iran. J. Med. Sci..

[6] Matsui H., Fushimi K., Yasunaga H. (2015). Variation in risk-standardized mortality of stroke among hospitals in Japan. PLoS ONE.

[7] Myint P.K., Bachmann M.O., Loke Y.K., Musgrave S.D., Price G.M., Hale R., Metcalf A.K., Turner D.A., Day D.J., Warburton E.A. (2017). Important factors in predicting mortality outcome from stroke: Findings from the anglia stroke clinical network evaluation study. Age Ageing.

[8] Nimptsch U., Mansky T. (2014). Stroke unit care and trends of in-hospital mortality for stroke in Germany 2005–2010. Int. J. Stroke.

[9] Reeves M.J., Bushnell C.D., Howard G., Gargano J.W., Duncan P.W., Lynch G., Khatiwoda A., Lisabeth L. (2008). Sex differences in stroke: Epidemiology, clinical presentation, medical care and outcomes. Lancet Neurol..

[10] El-Hajj M., Salameh P., Rachidi S., Hosseini H. (2016). The epidemiology of stroke in the Middle East. Eur. Stroke J..

[11] Almekhlafi M.A. (2016). Trends in one-year mortality for stroke in a tertiary academic center in Saudi Arabia: A 5-year retrospective analysis. Ann. Saudi Med..

[12] Barker-Collo S., Bennett D.A., Krishnamurthi R.V., Parmar P., Feigin V.L., Naghavi M., Forouzanfar M.H., Johnson C.O., Nguyen G., Mensah G.A. (2015). Sex differences in stroke incidence, prevalence, mortality and disability-adjusted life years: Results from the global burden of disease study 2013. Neuroepidemiology.

[13] Feigin V.L., Abajobir A., Abate K., Abd-Allah F., Abdulle A., Abera S., Abyu G., Ahmed M., Ärnlöv J., Vos T. (2017). Global, regional and national burden of neurological disorders during 1990–2015: A systematic analysis for the global burden of disease study 2015. Lancet Neurol..

[14] Al Khathaami A.M., Algahtani H., Alwabel A., Alosherey N., Kojan S., Aljumah M. (2011). The status of acute stroke care in Saudi Arabia: An urgent call for action!. Int. J. Stroke.

[15] Statistical Yearbook of 2016. https://www.stats.gov.sa/en/866-0.

[16] Okeng’o K., Chillo P., Gray W.K., Walker R.W., Matuja W. (2017). Early mortality and associated factors among patients with stroke admitted to a large teaching hospital in Tanzania. J. Stroke Cerebrovasc. Dis..

[17] Ryglewicz D., Barañska-Gieruszczak M., Lechowicz W., Hier D.B. (1997). High case-fatality rates in the warsaw stroke registry. J. Stroke Cerebrovasc. Dis..

[18] Goulart A.C., Bensenor I.M., Fernandes T.G., Alencar A.P., Fedeli L.M., Lotufo P.A. (2012). Early and one-year stroke case fatality in Sao Paulo, Brazil: Applying the world health organization’s stroke steps. J. Stroke Cerebrovasc. Dis..

[19] Van Asch C.J., Luitse M.J., Rinkel G.J., van der Tweel I., Algra A., Klijn C.J. (2010). Incidence, case fatality and functional outcome of intracerebral haemorrhage over time, according to age, sex and ethnic origin: A systematic review and meta-analysis. Lancet Neurol..

[20] Kalėdienė R., Rastenytė D. (2016). Trends and regional inequalities in mortality from stroke in the context of health care reform in Lithuania. Medicina.

[21] Alhazzani A.A., Mahfouz A.A., Abolyazid A.Y., Awadalla N.J., Aftab R., Faraheen A., Khalil S.N. (2018). Study of stroke incidence in the aseer region, Southwestern Saudi Arabia. Int. J. Environ. Res. Public Health.

[22] Thrift A.G., Howard G., Cadilhac D.A., Howard V.J., Rothwell P.M., Thayabaranathan T., Feigin V.L., Norrving B., Donnan G.A. (2017). Global stroke statistics: An update of mortality data from countries using a broad code of “cerebrovascular diseases”. Int. J. Stroke.

[23] Nkoke C., Lekoubou A., Balti E., Kengne A.P. (2015). Stroke mortality and its determinants in a resource-limited setting: A prospective cohort study in Yaounde, Cameroon. J. Neurol. Sci..

[24] Nadiah W.-A., Amir W.A., Muzaimi M., Mustafa M., Naing N.N. (2015). Determinants of mortality in first-ever stroke patients in the suburban Malaysia: A retrospective hospital-based study, 2005–2011. Iran. J. Public Health.

[25] Edjoc R.K., Reid R.D., Sharma M., Fang J. (2013). The prognostic effect of cigarette smoking on stroke severity, disability, length of stay in hospital and mortality in a cohort with cerebrovascular disease. J. Stroke Cerebrovasc. Dis..

[26] Xu L., Schooling C.M., Chan W.M., Lee S.Y., Leung G.M., Lam T.H. (2013). Smoking and hemorrhagic stroke mortality in a prospective cohort study of older Chinese. Stroke.

[27] Levine D.A., Walter J.M., Karve S.J., Skolarus L.E., Levine S.R., Mulhorn K.A. (2014). Smoking and mortality in stroke survivors: Can we eliminate the paradox?. J. Stroke Cerebrovasc. Dis..

[28] Liu Y., Yang Y., Jin H., Fan C., Lv P., Sun W., Peng Q., Zhao M., Jin D.K., Wang J. (2017). Discrepant relationships between admission blood pressure and mortality in different stroke subtypes. J. Neurol. Sci..

[29] Ntaios G., Faouzi M., Ferrari J., Lang W., Vemmos K., Michel P. (2012). An integer-based score to predict functional outcome in acute ischemic stroke the astral score. Neurology.

[30] Kelly J., Rudd T., Lewis R.R., Hunt B.J. (2002). Mortality from pulmonary embolism after acute stroke: Can we do better?. Age Ageing.

